# Effects of Dietary Resveratrol and Black Soldier Fly (*Hermetia illucens*) Larvae Meal Supplements on Quail Egg Production, Quality, and Consumer Acceptance

**DOI:** 10.3390/ani15010042

**Published:** 2024-12-27

**Authors:** Violeta Razmaitė, Artūras Šiukščius, Vidmantas Pileckas, Saulius Bliznikas

**Affiliations:** Animal Science Institute, Lithuanian University of Health Sciences, R. Žebenkos 12, LT-82317 Baisogala, Lithuania; arturas.siukscius@lsmu.lt (A.Š.); vidmantas.pileckas@lsmu.lt (V.P.); saulius.bliznikas@lsmu.lt (S.B.)

**Keywords:** resveratrol, black soldier fly, quail, egg production, egg quality, healthiness, consumer

## Abstract

This study aimed to examine the effects of supplementing a basal diet with resveratrol and black soldier fly (*Hermetiaillucens*) larvae meal on Manchurian Golden quail egg production and quality as well as consumer attitudes towards the quail eggs and their acceptability. Quail were allotted three treatments for a laying period of 3 months. The dietary treatment groups were those of a basal diet, a basal diet with 250 mg/kg resveratrol pliusACE, and a diet supplemented with 10% black soldier fly larvae meal (BSF). The BSF larvae meal supplement increased the live weight of the quail, but the differences in the mean egg production and egg external parameters between the experimental groups were insignificant. The egg yolk from the quail fed with resveratrol and BSF supplementation demonstrated lower (*p* < 0.001 and *p* < 0.01) pH compared with the quail fed a basal diet. Egg yolk colour showed lower (*p* < 0.05) lightness and lower (*p* < 0.01) yellowness and colour saturation in the group of quail fed with resveratrol supplement compared with BSF supplement. Resveratrol inclusion in the basal diet resulted in increased (*p* < 0.001) protein content in the yolk. BSF inclusion in the diet showed the effect by increasing (*p* < 0.001) dry matter in albumen. The total monounsaturated fatty acids, polyunsaturated fatty acids, and cholesterol content were not affected by the dietary treatment. The inclusion of BSF larvae meal in the diet increased the proportion of total saturated fatty acids and lowered the proportion of total *trans* fatty acids in yolk lipids and demonstrated less favourable ratios of fatty acids and nutritional indices, except for the peroxidisability index, and showed higher oxidative status (*p* < 0.05), flavour score, and overall egg acceptance compared with the quail fed on a basal diet.

## Simple Summary

The egg is an inexpensive and well-balanced food source of quality nutrients. While most eggs consumed nowadays are chicken eggs, the eggs of quail are more and more often available. At the same time, consumer demands for quality and diversity of food are growing as living standards improve. Moreover, increased interest is also observed in the use of products that may help to improve the quality and sustainability of poultry production and reduce environmental pollution. Among the products with alternative sources of protein for poultry, including quail, are insects and compounds containing bioactive phytochemicals. As there is a great variety in quail breeds and lines and in insect meal products and bioactive phytochemicals, the effects of resveratrol and defatted black soldier fly (*Hermetia illucens*) larvae meal inclusion in the diets of Manchurian Golden quail have been studied.

## 1. Introduction

Quail, due to their small size, rapid growth, high productivity, and relatively low raising costs, are well suited to both small flock and commercial production, and are one of the fastest growing animal industries. They have been introduced in many countries; however, until now they are raised mainly in Asia and remain regional preferences for quail production [1,2,3]. Although complete quail egg production and consumption data remain limited for many European countries, and quail eggs are still considered an exotic and gourmet food [4,5], they are becoming more common in European cuisines. Quail eggs can be prepared in the same manner as chicken eggs: poached, hard or soft boiled, fried, scrambled, or used as an ingredient or garnish in different dishes [1,6]. Quail eggs are nutrient dense and contain superior origin of protein, lipids, vitamins, and minerals to humans, as well as a number of other factors to protect against bacterial and viral infections [6,7]. There are studies that have suggested that egg consumption can protect individuals against metabolic syndrome (MetS) by increasing HDL-C levels and reducing inflammation, but on the other hand, the egg contains a lot of cholesterol. This has led to the perception that eggs can lead to cardiovascular disease (CVD) risks and other problems. However, published data show that the effect of egg yolks on serum cholesterol levels and some health problems is still controversial [5,7]. However, some comparative studies showed that there was no significant difference in cholesterol level between chicken and quail eggs in terms of the yolk [5], whereas other studies demonstrated that quail yolk lipids contain a favourable polyunsaturated and saturated (PUFA/SFA) ratio and displayed the lowest fat and cholesterol content and the lowest values for the cholesterol index (CI) and cholesterol–saturated fat index (CSI) compared with different avian species [4]. Therefore, further research is being carried out to increase quail production and improve the quality of quail eggs.

Resveratrol is a natural plant polyphenol and possesses antioxidant, antimicrobial, anti-inflammatory, and other qualities [8,9].Therefore, different levels of resveratrol as a feed additive have been studied in animal production, including poultry. There are studies that have shown that dietary resveratrol improves growth performance, meat and egg quality and alleviates the adverse effects induced by heat stress [9]. A review of the literature sources has shown that resveratrol may behave as an antioxidant or pro-oxidant depending on many parameters, including the dose and microenvironment. Moreover, resveratrol has biphasic concentration-dependent effects, being an antioxidant at low doses and pro-oxidant at high doses [9]. The research on resveratrol supplementation to improve production has long been controversial [10,11,12].

Like for other farm animals, plant-derived feeds comprise the main ingredients of quail feed. However, animal proteins are also needed to maintain superior amino acids. As animal proteins are a limited alternative, protein sources of comparable value are needed for poultry production [13]. Insects are among the most numerous and abundant animal species on the planet. Owing to their abundance and nutritional value, they are currently regarded as the most promising and sustainable source of animal protein [14]. Moreover, insects are the natural food of most birds; thus, the use of fly larvae for feed production has a biological basis, and, therefore, the potential of insect protein in quail diets has received attention [13,15,16,17]. Among different insect species, black soldier fly (*Hermetia illucens*) larvae and mulberry silkworm (*Bombyx mori*) chrysalis meals are of great interest for quail feeding, and many positive indications of the insect use have been reported [16,18]. The nutritional value of black soldier fly (*Hermetia illucens*) larvae (BSF) is considered to have a good profile of essential amino acids (EAAs), with leucine, lysine, valine, and histidine being the dominant EAAs. Minerals such as calcium, potassium, phosphorus, sodium, magnesium, zinc, iron, manganese, and copper were found to be in adequate amounts. Vitamins B1, B2, and C were also present in the BSF. [19] However, there are still many controversial data and unanswered questions. Therefore, further studies should investigate the carryover effects of dietary resveratrol and fly larvae supplementation on product quality with respect to shelf life, antioxidant stability, and nutritive value for human consumption.

This study aimed to examine the effects of supplementing a basal diet with resveratrol and black soldier fly (*Hermetia illucens*) larvae meal on Manchurian Golden quail egg production and quality as well as consumer attitudes towards quail eggs and acceptability.

## 2. Materials and Methods

### 2.1. Experimental Design

Quail egg laying and quality experiment was conducted in accordance with the Directive 2010/63 EU of European Parliament and of the Council of 22 September 2010 on the protection of animals used for scientific purposes [20] and approved by the Institutional Board of the Animal Science Institute of the Lithuanian University of Health Sciences (protocol No 24/01/30/01) on 30 January 2024.

A total of 150 Manchurian Golden quail were allotted to 3 treatments for a laying period of 3 months. The dietary treatment groups were those of a basal diet;a basal diet with 250 mg/kg resveratrol plius ACE (Symbiofarm Sp.zo.o, Czudec, Poland), which contained 98% standardised extract of resveratrol derived from *Polygonum cuspidatum;* and a diet supplemented with 10% of black soldier fly (*Hermetia illucens*) defatted larvae meal (BSF).After starting to lay eggs at 36 days of age, the quail continued to receive the grower diets for up to 8 weeks of age. From week 8, the diets were changed. The diets were formulated to meet the requirements for quail [21,22] and to be as isonitrogenous as possible (Table 1). The diets and the nutritional value of feed mixtures were calculated using ration software HYBRIMIN^®^ Futter 5 (https:hybrimin.com/hybrimin-futter-5/, accessed on 25 December 2024). The feeds and water for all the groups were provided ad libitum.

BSF larvae meal inclusion increased proportion of saturated fatty acids and decreased proportion of polyunsaturated fatty acids in feed mixture (Table 2).

Chemical composition of BSF used in feed mixture is presented in Table 3.

The quail were housed indoors in Cimuka Comfortplast cages (5–6 quail per cage) at an average temperature of 23.3°C (19–27°C). There were 9 replicates per treatment. The cages were fully equipped with feeders and nipple drinkers. Each bird was provided with, on average, 371 cm^2^ floor space. The quail started to lay at 36 days of age when the natural lighting was L12.5 h: D11.5 h, and the eggs for evaluation were collected until midsummer, when the natural lighting increased to L17.4 h: D6.6 h.

### 2.2. Evaluation of Quail Productive Performance

Prior to their laying cycle and at the end of the experiment, the quail were individually weighed using an electronic balance (Model KB 600, Romasas, Šiauliai, Lithuania) with an accuracy of 0.1 g to monitor their live weight change during the experiment. Laying quail mortality was recorded throughout the experiment. During the experiment, all laid eggs, including defective ones, were counted daily. Egg production was determined as the number of laid eggs on the number of laying quails × 100. Defective eggs (without solid shell and broken) were also counted daily, and the percentage of the total laid eggs was calculated. Five weeks post the start of laying, the eggs were collected from each group to determine their physicochemical and sensory qualities and storage stability. A total of 1683 eggs (561 per treatment) were used for the quality evaluation. Supplementary Materials regarding the numbers of eggs and their analysis can be found in Table S1.

### 2.3. Evaluation of Egg Physical Characteristics

A total of 306 eggs was used to evaluate external and internal egg quality characteristics. The eggs were weighed using an electronic balance (Model ATZ 220, AXIS Sp.zo.o, Gdansk, Poland) with an accuracy of 0.001 g. Egg equatorial diameter (mm) and egg height (mm), using a steel Vernier calliper of 0.05 mm accuracy (0–160 mm—CCCP, no 9712912), were measured and used to calculate the shape index: equatorial diameter/height ×100. The components of these eggs after boiling for 3 min, including albumen, yolk, and shell, were weighed separately. The pH of the boiled yolk was determined using a digital portable FiveGoTM pH meter F2 (MettlerToledo GmbH, Schwerzenbach, Switzerland) equipped with a Mettler Toledo pH electrode LoT406-M6-DXK-S7/25 with a Xerolyt polymer electrolyte. In order to form a sufficient amount of material to perform colour measurements, the yolks of six eggs were homogenised to one sample, and colour of 17such samples was determined in the CIE L* a* b* and L* C h colour spaces system using Minolta CR-410 colorimeter (Konica Minolta, Osaka, Japan) equipped with a C illuminant and 2° standard observer calibrated to a standard white calibration plate (Y = 85.3, x = 0.3173, y = 0.3251).

### 2.4. Chemical Analyses

#### 2.4.1. Chemicals Used for Analyses

Sulphuric acid, hydrochloric acid, boric acid, sodium hydroxide, and Kjeltabs ST were used for crude protein content determination; petroleum ether (b.p. 40–60 °C) and sodium sulphate anhydrous were used for crude fat content determination; trichloroacetic acid, 2-thiobarbituric acid, potassium dihydrogen phosphate, EDTA, propyl gallate, 1,1,3,3-tetraethoxypropan, methanol, and acetonitrile were used for malondialdehyde (MDA) determination; 2-propanol, acetonitrile, hexane, potassium hydroxide, and cholesterol were used for cholesterol content determination; hexane, methanol, chloroform, sodium methoxide solution, potassium chloride, hydrochloric acid and fatty acid standards “37 Component Fame Mix”, and trans fame mix k 110 were used to determine fatty acid profiles. All these reagents except Kjeltabs ST (VelpScientifica, Via Stazione, Italy) and fatty acid standards (Supelco Analytical, Bellefonte, PA, USA) were purchased from (Sigma-Aldrich Co., St. Louis, MO, USA).

#### 2.4.2. Egg Proximate Composition

To obtain enough material to perform the analysis, the content of eight boiled eggs was homogenised for each sample. Therefore, a total of 360 eggs was collected and used for chemical composition and egg yolk lipid profile analyses. The dry matter content was determined [23] by drying samples in an oven at 105 °C until a constant weight was obtained (method No. 950.46B; AOAC, 1990). The crude protein content was determined by the Kjeldahl method using the VelpScientifica equipment (VelpScientificasrl., Usmate, Italy), and a conversion factor of 6.25 was used to convert total nitrogen to crude protein (method No. 981.10; AOAC, 1990). Crude fat was determined by the Soxhlet extraction method (method No. 960.39; AOAC, 1990) by using Soxtherm apparatus (Gerhard GmbH &Co KG, Königswinter, Germany). Ash was determined by incineration in a muffle furnace at 550 °C for 24 h (method No. 920.153; AOAC, 1990). The samples were analysed in duplicate for all analytes. The content of protein, fat, and ash were expressed as the weight percentage of dry matter.

The cholesterol content was determined in 45 (15 from each group) raw yolk samples according to the extraction method described by Zhang et al. [24] and was followed by HPLC separation and analysis on Shimadzu HPLC system (Shimadzu Corp., Kyoto, Japan), which included a system controller SCL-10A, solvent delivery module LC-10AT, auto injector SIL-10AD, UV-Vis detector SPD-10AV, column oven CTO-10AC, and on-line degasser DGU-14A. The data collection and evaluation were performed by using LC Solution (Shimadzu Corp., Kyoto, Japan) operating system. The analytical column was LiChrospher 100 RP-18e, 150 × 4.6 mm, 5 µm(Alltech Associates Inc., Columbia, MD, USA) with a guard column (LiChrospher 100 RP-18, 7.5 × 4.6 mm). The cholesterol content was expressed as mg/g wet yolk weight.

#### 2.4.3. Determination of Fatty Acid Profiles

The extraction of lipids for fatty acid analysis was performed in 45 samples (15 from each group) with a mixture of 2volumes of chloroform and 1volume of methanol as described by Folch et al. [25]. Methylation of the samples was performed using sodium methoxide: 5 mL of 25 wt % solution in methanol was added to the sample and stirred. After 1 h, 7 mL HCL, 6 mL hexane, and 2 mL H_2_O were added. The top layer was transferred into a new test tube and evaporated. Fatty acid methyl esters were prepared according to the procedure described by Christopherson and Glass [26]. The FAMEs were analysed using a gas chromatograph (GC-2010 PLUS, Shimadzu Corp., Kyoto, Japan) fitted with a flame ionisation detector. The separation of the methyl esters of fatty acids was affected on the capillary column Rt 2560 (100 m × 0.25 mm × 0.2 μm; Restek, Bellefonte, PA, USA) by temperature programming from 160 °C to 230 °C. The temperatures of the injector and detector were held at 240 °C and 260 °C, respectively. The rate of flow of carrier gas (nitrogen) through the column was 0.79 mL/min. The peaks were identified by comparison with the retention times of the standard fatty acid methyl esters “37 Component FAME Mix” and *trans* FAME MiX k 110 (Supelco Analytical, Bellefonte, PA, USA). The relative proportion of each fatty acid was expressed as the relative percentage of the sum of the total fatty acids using LAB Solutions LC/GC (version 5.71) software for Shimadzu gas chromatograph workstations.

#### 2.4.4. Storage Stability of Eggs

The egg storage stability was tested on 90 eggs. Ten out of thirty eggs per each treatment were analysed on the day of their collection, and the rest were stored in closed containers at +4 °C until their analysis on storage day 30 and day 60 to measure the malondialdehyde (MDA) contents. MDA in raw yolks was extracted according to the procedure of Mendes et al. [27] and analysed chromatographically on HPLC system Shimadzu 10 A_VP_ (Shimadzu Corp., Kyoto, Japan). HPLC system consisted of a solvent delivery module LC-10AT_VP_, auto injector SIL-10AD_VP_, spectrofluorimetric detector RF-10A_XL_, column oven CTO-10AC_VP_, low-pressure gradient flow control valve FCL-10AL_VP_, solvent degasser DGU-14A, and system controller SCL-10A_VP_. Data collection and evaluation wereperformed by using LC Solution (Shimadzu Corp., Kyoto, Japan) operating system. The analytical column was LiChrospher 100 RP-18, 250 × 4.6 mm, 5 μm (Alltech Associates Inc., Chicago, IL, USA) with a guard column (LiChrospher 100 RP-18, 7.5 × 4.6 mm). The mobile phase consisted of 50 mM KH_2_PO_4_ buffer solution, methanol, and acetonitrile in the proportion 72:17:11 (*v/v*) and pumped isocratically at a flow rate of 1.0 mL/min. Spectrofluorimetric detector wavelengths were set at 525 nm (excitation) and 560 nm (emission). The results were expressed as μmol MDA present in 1 kg of yolk.

### 2.5. Lipid Quality Indices

Lipid quality indices, i.e., atherogenic index AI = [C12:0 + (4 × C14:0) + C16:0]/ΣUFA and thrombogenic index TI = [C14:0 + C16:0 + C18:0]/[0.5 × MUFA+0.5xn-6PUFA + 3x n-3 PUFA + n-3 PUFA/n-6 PUFA], were calculated according to Ulbricht and Southgate [28,29]. The hypocholesterolemic/hypercholesterolemic ratio modified by Merliță [30] h/H = [(cis C18:1n-9 + C18:1n-7 + PUFA]/[C12:0 + C14:0+C16:0] and health-promoting HPI = [UFA/C12:0 + C14:0 + C16:0] index were calculated as described by Chen and Liu [29], and the flesh lipid quality FLQ = [100 × (C20:5n-3 + C22:6n-3)/SFA] index was calculated as described by Atia et al. [31]. The peroxidisability index PI = [(C14:n-7 + C16:1n-9 + C16:1n-7 + C17:1n-9 + C18:1n-9t + C18:1n-9 + C18:1n-7 + C20:1n-9) × 0.025] + [(C18:2n-6t,c + C18:2n-6) × 1] + [(C18:3n-3 + C18:3n-6 + C20:3n-6) × 2] + [(C20:4n-6 + C22:4n-6) × 4]+[(C20:5n-3 + C22:5n-3) × 6] + [C22:6 × 8] was determined according to Du et al. [32].

### 2.6. Consumer Sensory Evaluation and Emotional Response to Quail Eggs

With the aim to evaluate quail egg consumption and attitudes in addition to preferences regarding total egg consumption, a paper questionnaire-based survey and six repeated consumer panel sessions on different days were conducted. A total of 132 individuals participated in the consumer study. The characteristics of the participants in the consumer survey and egg sensory test are presented in Table 4. The participants varied in sociodemographic characteristics, individual attitudes, and criteria. A higher proportion (67.4%) of participants were women because there are more women in Lithuania, and they are more often interested and responsible for food in the families.

The consumers were mainly recruited from the staff members of the Lithuanian University of Health Sciences and from the communities around the Kaunas and Baisogala areas. All the respondents were egg consumers. The participants were supplied with a ballot, knife, cup, napkins, water, and palate cleansers (unsweetened tea) to use between eggs. Before the start of each panel, the participants were given verbal instructions regarding the ballot and usage of the palate cleansers. Primarily, each respondent completed the questionnaire. The questionnaire assessed the following sociodemographic variables: gender, year of birth, and education;it included questions related to the frequency of egg consumption and consumer preferences in choosing the type of eggs and the main reasons that determine their choice. The questionnaire also included questions about quail egg consumption and changes related to the consumption of quail eggs. After the respondents had completed the questionnaire, they were asked to evaluate quail eggs. Blind liking (perceived sensory acceptability) was used for the evaluation of eggs. The eggs for each session were collected daily and boiled for 3 min. During the sensory test, consumers received coded whole warm unpeeled quail eggs and were asked to peel the eggs themselves, taste them in a pre-defined random order, and detect possible differences between the dietary treatments. The participants were asked to score aroma, colour intensity of yolk, flavour/taste, and overall liking of the eggs on a nine-point scale (from 1 = dislike extremely to 9 = like extremely) according to their perceived values and overall acceptability. The panel sessions lasted for about 1 h.

Additionally, to assess the emotional response to quail eggs of quails from different dietary treatments using FaceReader software (https://www.noldus.com/facereader, accessed on 23 December 2024), 90 daily collected eggs were sent to the Veterinary Academy. Boiled eggs were evaluated by trained participants from a sensory assessment using FaceReader software (Noldus Information Technology, Wageningen, The Netherlands) connected to a web camera (Microsoft Corporation, Redmond, WA, USA) to assess the expressions of participant emotions (by viewing, sniffing, and tasting the eggs) as described by Nutautaitė et al. 2019 [33].

### 2.7. Statistical Analysis

The data were subjected to the analysis of variance in the general linear (GLM) procedure in IBM SPSS Statistics 29 for Windows, Version 29 Armonk, NY, USA: IBM Corp software with LSD tests to determine the significance of differences of estimated marginal (EM) means between the groups. The GLM model included the fixed factor of the feeding group (basal diet, basal diet supplemented by resveratrol, and diet supplemented by BSF larvae meal). Descriptive analyses were performed on egg consumption frequencies and consumer attitudes and preferences regarding eggs. The liking data were subjected to the analysis of variance in repeated measures of the general linear (GLM) procedure. For the effect of consumer gender and generation/age evaluation, the GLM univariate model included fixed factors of consumer gender and generation. The differences were regarded as significant when *p* < 0.05.

## 3. Results and Discussion

### 3.1. Productive Performance of Laying Quail

The dietary treatment affected the productive performance of laying quail (Table 5). 

The increased average live weight of the quail fed with BSF larvae meal supplement after the 3-month laying period was 37.5 and 43.2 g higher (*p* < 0.05) compared with the quail fed on the basal diet and resveratrol, respectively. BSF larvae meal is a rich source of not only animal origin protein but also of fat and energy that increased the growth of young laying quails in the present study. In contrast to our study, the results of other authors [16] who in their experiments used older (six months of age) quail, showed insignificant decrease in the final live body weight of laying quail. However, the obtained results of resveratrol supplementation in the present study agree with the findings of other authors [10], who reported no significant resveratrol supplementation effects on the body weight gain for growing quails. The mortality of the quails was low (0.05–0.16%) and not affected by the dietary treatment. The quail fed with resveratrol supplement showed 5.2 and 5.4% lower (*p* < 0.001) egg production in the second laying month than the quail fed with BSF larvae meal supplement and the basal diet, respectively. The highest egg production of quail fed on a basal diet and resveratrol supplement was recorded in the third month of laying, but the differences between the groups had decreased. The quail fed with BSF larvae meal and resveratrol supplementation demonstrated 3.0 and 4.4% lower (*p* < 0.05) egg production, respectively, compared with the quail fed on a basal diet. The differences in the mean egg production between the experimental groups in all three laying months were insignificant, and this is in agreement with the findings of other authors for resveratrol [10] and BSF larvae meal inclusion in quail diets [16]. The inclusion of full-fat silkworm meal in the diet for laying quail demonstrated an increase in egg production [18], while after the use of resveratrol supplementation for hen feeding, different authors reported contradictory results on egg production [34,35,36]. The egg shell quality is very important for the protection of the egg content from mechanical impacts and microbial invasion and may result in lowering egg losses before the eggs arrive at retail [37]. In the present study, the percentage of defective eggs was not affected by the dietary treatment, and this agrees with the findings of other authors [16]. The higher percentage of defective eggs was found only at the beginning of the laying period.

### 3.2. Egg External and Internal Characteristics

The weight of the evaluated eggs from Manchurian Golden quail under different dietary treatments in the present study was similar; there were no significant differences in their external parameters such as egg height, egg diameter, and egg shape index between the groups (Table 6).

The egg shell weight and shell percentage of quail fed with BSF larvae meal supplement was higher (*p* < 0.05) than those of quail fed with resveratrol supplement. It seems that the quail demonstrated a slightly higher calcium assimilation from the feed supplemented with BSF larvae meal. The albumen weight in the eggs of quail fed with BSF was lower (*p* < 0.05) compared with both other groups, and the albumen percentage was also lower (*p* < 0.05 and *p* < 0.01) than in the eggs of quail fed on a basal diet and resveratrol supplement, respectively. Significant differences were reported for egg weight, shape index, shell, albumen, and yolk weights among the different lines and phenotypes of quail [38,39]. A similar increase in shell weight and shell percentage related to the use of BSF larvae meal in the laying quail diet was observed in the experiment of Dalle Zotte et al. [16]. Dietary and housing effects on external qualities, including shell weight and ratio of quail egg, were reported by Hossain et al. [40]. 

The yolk of boiled quail eggs demonstrated lower pH than the yolk of raw eggs in our previous study [41]. The yolk of eggs from the quail fed a basal diet demonstrated higher (*p* < 0.001 and *p* < 0.01) pH compared with the quail fed resveratrol and BSF supplementation (Table 7).

Egg yolk colour in the CIE Lab system showed lower (*p* < 0.05) lightness and lower (*p* < 0.01) yellowness and colour saturation chroma but higher (*p* < 0.05) hue angle in the group of quail fed with resveratrol supplement compared with BSF supplement. In our previous study [41], the yolks of raw eggs from the quail fed with rapeseed hemp and camelina supplementation exhibited lower lightness and higher greenishness, yellowness, and chroma than in the present study using the basal diet and resveratrol and BSF larvae meal supplementation.

### 3.3. Proximate Composition

The proximate composition of the quail egg yolks, albumen, and whole edible portions was affected by the dietary treatment (Table 8). Resveratrol inclusion into the basal diet increased (*p* < 0.001) the protein content in yolks. The yolks of eggs from the quail fed with BSF supplementation had lower (*p* < 0.01) lipid content compared with the quail fed a basal diet. The cholesterol content in the yolks was not affected by the dietary treatment with either resveratrol or BSF larvae meal supplementation. Although some authors [4] reported that quail yolks displayed the lowest fat and cholesterol content and the lowest values for the cholesterol index and cholesterol–saturated fat index compared with other poultry species anyway, quail eggs are characterised by high cholesterol content in their yolks, and there are efforts to reduce cholesterol using ginger with probiotics [42] and dietary resveratrol [33] or even to use cholesterol removal treatment [43]. Lu et al. [44] reported that high supplementation of BSF larvae meal can increase concentrations of cholesterol and triglycerides. However, the content of cholesterol was found to be slightly lower than that determined using hemp (12.94 mg/g) and camelina (13.18 mg/g) supplementations in our previous study [41].

In addition, resveratrol inclusion into the basal diet increased (*p* < 0.01) dry matter and ash in albumen. BSF inclusion in the diet showed the effect of increasing the dry matter content in albumen (*p* < 0.001) and the whole edible portion (*p* < 0.05) and of ash in all edible parts of the egg. The obtained results confirmed the published results of other authors [40], who reported that changes in quail diet can have a substantial impact on egg proximate composition. 

### 3.4. Fatty Acid Profiles

The inclusion of BSF larvae meal in the diet increased (*p* < 0.05 and *p* < 0.01, respectively) the proportion of total saturated fatty acids (SFA) in yolk lipids compared with yolk lipids from the quail fed on a basal diet and resveratrol supplementation (Table 9) because the content of saturated fatty acids in BSF larvae exceeded that of the unsaturated fatty acids [19], and the diet with BSF larvae meal in the present study had a relatively (9.7%) higher proportion of saturated fatty acids compared with the basal diet. Of all saturated fatty acids,the one undesirable in the human diet, myristic (C14:0) fatty acid, increased (*p* < 0.001) by as much as 83.8%. The effects of BSF larvae meal supplementation on yolk SFA was similar to those reported by Dalle Zotte et al. [16].

Despite the proportion variations in individual monounsaturated and polyunsaturated fatty acids between the groups, the total monounsaturated fatty acids (MUFAs) and total polyunsaturated fatty acids (PUFAs) were not affected by the dietary treatment. The proportion of total *trans* fatty acids, the intake of which may harm human health, including both trans-MUFA and trans-PUFA, was lower (*p* < 0.01) in the yolk of quail fed with BSF larvae meal supplementation. Different fatty acids can play a positive or a negative role in terms of human health and disease prevention; therefore, efforts are being made to determine their nutritional value. The nutritional indices and ratios of fatty acids can help to assess the nutritional value of fatty acids [29].

A lower (*p* < 0.05) and less favourable PUFA/SFA ratio was detected in the yolk lipids of quail receiving feed supplemented with BSF larvae meal (Table 10). 

The PUFA/SFA ratios in other groups were above the minimum (0.4) recommended for the diet [43,45], and the higher the ratio, the more positive the effect [29]. The n-6/n-3 PUFA ratios in the yolk were high in all groups and did not meet the recommendations of Bellagio’s report on healthy agriculture, healthy nutrition, and healthy people, which indicated that a4:1ratio of n-6 PUFA to n-3 PUFA in the diet should be the goal [46]. BSF larvae meal supplementation resulted in increasing(*p* < 0.01) this ratio compared with the basal diet. BSF larvae meal supplementation also increased atherogenic (AI) and thrombogenic (TI) indexes, which characterise the atherogenic and thrombogenic potentials of fatty acids, respectively. Both AI and TI indexes can be used to assess the potential effects of fatty acid composition of food. Although no organisation has yet provided the recommended values for the AI and TI [29], the egg yolk with a lower AI and TI can be considered as having a better nutritional quality, and its consumption may reduce the risk of coronary heart disease (CHD). The highest and most favourable h/H ratio, which characterises the relationship between hypocholesterolemic fatty acids and hypercholesterolemic fatty acids, was detected in the yolk lipids of the quail fed with resveratrol supplementation. The h/H ratio in the lipids of quail fed with BSF larvae meal supplementation was lower (*p* < 0.001 and *p* < 0.05) compared with resveratrol supplementation and a basal diet, respectively. A worse n-6/n-3 PUFA ratio and AI and TI indexes when using BSF larvae meal were also reported by Dalle Zotte [16]. The health-promoting index (HPI) and flesh lipid quality index (FLQ) are less common for the evaluation of egg lipids [30]. The lowest (*p* < 0.001 and *p* < 0.01) values of health-promoting index (HPI) and flesh lipid quality index (FLQ) were also estimated in the yolk lipids of the quail fed with BSF larvae meal supplementation. The HPI is the inverse of the AI, and the FLQ shows the sum of EPA and DHA as a percentage of total FA [28] or SFA [30]; therefore, food with higher HPI and FLQ values is assumed to be more beneficial to human health. However, a lower (*p* < 0.05) and more favourable peroxidisability index (PI) was detected in the yolk lipids of the quail fed with BSF larvae meal supplementation.

### 3.5. Egg Storage Stability

Inclusion of BSF larvae meal in the diet of laying quail tended to show slightly lower (*p* = 0.065) content of malondialdehyde (MDA) in the yolk of freshly laid raw eggs compared with the eggs of the quail fed on a basal diet (Table 11).

This finding coincides with lower peroxidisability indexes (PIs) calculated for the same groups (Table 10). Malondialdehyde (MDA) is an important compound and is widely used as a marker of lipid peroxidation [47,48]. Sahin et al. [10] have reported that the addition of resveratrol decreased the egg yolk MDA concentration; however, the MDA concentration decreased linearly in response to the increasing dietary resveratrol level, and inclusion of resveratrol up to 400 mg/kg into quail diets enhanced the antioxidant status of eggs. In the present study, inclusion of 250 mg/kg resveratrol in the diet did not show a significant (*p* > 0.05) decrease in MDA compared with the basal diet and BSF larvae meal inclusion. Considering that resveratrol has biphasic concentration-dependent effects, being an antioxidant at low doses and pro-oxidant at high doses [9], it remains unclear what dose would be appropriate. During the storage period of 30 days at 4 °C temperature, the content of MDA increased (*p* < 0.001) from 2.1 times in the yolk of quail fed with resveratrol supplementation to 2.2 times in the yolk of quail fed on a basal diet. The difference in MDA content in the yolk of the quail fed on a basal diet being 31.5% higher (*p* = 0.065) compared with BSF larvae meal supplementation at day 0 increased to 33.1% (*p* < 0.05) at day 30. MDA increase during quail and chicken eggs’ storage was also found by other authors [16,47,49]. Although further storage of eggs after 30 days did not influence the amount of MDA, it cannot be stated that quail eggs are suitable for longer storage based only on the determined MDA.

### 3.6. Consumer Surveys

Diversity of egg consumption frequences, attitudes to egg properties, and choice criteria were analysed in the respondent answers using the questionnaire survey. The respondents reported that most frequently (47%) they consume eggs two–three times weekly. Consumption of eggs four–six times weekly and daily was reported by 8.3% and 6.8% of respondents, respectively. However, 31.1% of respondents reported that they consume eggs rarely or as an ingredient in other dishes (6.8%). Although all the respondents answered that usually they use hen eggs, 19.1% of them indicated changes in their choice of eggs and increasing consumption of quail eggs; however, 35.1% of respondents admitted that they had never eaten quail eggs. Taste, freshness, size, and the effect on consumer health were mentioned as the most important egg qualities (48.5%, 13.6%, 13.6%, and 12.9%, respectively).

### 3.7. Consumer Sensory Evaluation

There were no significant differences in the blind evaluation of quail egg odour between the treatment groups (Table 12). Colour intensity of the yolk from the quail fed with a BSF larvae meal supplement had a higher (*p* < 0.01) score compared with the eggs from the quail fed on a basal diet and resveratrol supplementation, and this is in agreement with the highest yolk yellowness and chroma values obtained using instrumental colour evaluation for the eggs from the same groups. Moreover, it is assumed that *H. illucens* adult flies can be a useful source of natural pigments with antioxidant properties [50] because insects, including the black soldier fly (BSF), have different chemical pigments, including carotenoids, ommochromes, and others [51]. Therefore, it is likely that their larvae also have pigments that could affect the yolk colour.

The taste of the eggs from the quail fed with BSF larvae meal supplement was also scored higher (*p* < 0.01) as was (*p* < 0.05) the overall egg acceptance compared with the quail fed on a basal completely plant-based diet. Meanwhile, other authors [16] did not find an effect of the inclusion of BSF larvae meal in the feed on quail egg sensory attributes by employing trained panellists. As other authors [52] have reported, consumer acceptance of insects as a feed ingredient is very important both for the development of the sector of insect farming and the products obtained from animals fed an insect-based diet. Moreover, providing information on the sustainability and nutritional benefits of using insects as a feed increased both the attitude and intention to purchase and use the products made from animals fed with insects [52,53,54]. The gender and generation of the participants did not affect the acceptance of the tested quail eggs.

### 3.8. Emotional Response 

Seven emotional states were classified by Noldus’s FaceReader. There were no significant differences in the emotional response to quail eggs in view, odour, or taste between the treatment groups (Table 13). As the FaceReader software provides the values for the emotional states ranging from 0 to 1 and the valence range from −1 to 1 [33,55,56], it could be considered that the most pronounced emotion to the eggs was neutral. Four emotional states (sad, angry, scared, and disgusted) were classified as negative by Noldus’s FaceReader, and happiness as a positive emotion, whereas surprise and contempt can be assigned to these categories only depending on the context [56]. Emotional valence, calculated as a quantitative measure that assesses the emotion’s polarity from positive to negative, was slightly negative in all groups. The biggest difference between the treatment groups was found in sadness expression for egg taste. The sad emotion was highest when evaluators tasted eggs from the quail group fed the basal diet. This can be considered to be equivalent to the taste evaluation during the consumer test, when the taste of eggs from the group fed with the basal diet was evaluated with a slightly lower score. Evaluations with questionnaires might not always be sufficient to measure initial emotions, and the use of facial recognition software is becoming increasingly relevant. Although the use of FaceReader software is a good tool for measuring emotions and is significantly less time-consuming than questionnaire-based measures, Landmann [56] reviewed the limitations and limiting factors of both these methods and pointed out that, together with other measurement methods in a multimethod approach, the validity of the results can be strengthened.

## 4. Conclusions

The results obtained in this study revealed the highest and lowest increase in live weight of the laying quail fed with BSF larvae meal and resveratrol supplements, respectively. The diets supplemented with resveratrol and BSF larvae meal did not affect the egg production. The diet supplemented with BSF larvae meal increased the shell weight and proportion in the egg. However, inclusion of 250 mg/kg resveratrol in the diet increased the protein content in the yolk. Supplementation of 10% BSF larvae meal in the diet of laying quail increased the proportion of saturated fatty acids and demonstrated less favourable lipid quality indices of the yolk. However, BSF larvae meal inclusion improved oxidative stability during storage and showed good acceptance by consumers. A consumer test can be a good tool both for assessing consumer preferences and attitudes towards eggs laid by quail fed different diets and also for information dissemination about egg quality and increased intensions to consume quail eggs, including those from insect-fed quail. The data of the study provides evidence that additional investigations are required for determination of the most appropriate amounts of resveratrol and BSF larvae meal inclusion in the diets of laying quail.

## Figures and Tables

**Table 1:** Composition of quail feed and its nutritive value.

Ingredients	Feed for Quail Up to 8 Weeks of Age and Treatment Groups			Feed for Quail from 8 Weeks of Age and Treatment Groups		
	Basal Diet	Resveratrol Suppl.	BSF Suppl.	Basal Diet	Resveratrol Suppl.	BSF Suppl.
Maize, %	25.28	25.28	27.24	22.70	22.70	24.65
Wheat, %	28.00	28.00	28.00	25.00	25.00	25.00
Soya meal, %	37.00	37.00	25.00	30.00	30.00	18.00
Sunflower meal, %	3.00	3.00	3.00	10.00	10.00	10.00
Rape oil, %	2.00	2.00	2.00	2.00	2.00	2.00
Fodder limestone,%	2.00	2.00	2.00	8.00	8.00	8.00
Monocalcium phosphate, %	1.00	1.00	1.00	0.80	0.80	0.80
Premix for rearing layers, %	1.00	1.00	1.00	1.00	1.00	1.00
Fodder salt, %	0.35	0.35	0.35	0.35	0.35	0.35
Lysine HCL, %	0.20	0.20	0.27	-	-	0.10
Methionine, %	0.17	0.17	0.14	0.15	0.15	0.10
Resveratrol, mg/kg	-	+250	-	-	+250	-
Black soldier fly larvae meal, %	-	-	10.00	-	-	10.00
Calculated nutritional value of feed mixture						
Dry matter, %	87.84	87.84	88.65	88.66	88.66	89.46
Metabolizable energy (ME), MJ/kg	11.15	11.15	10.83	10.32	10.32	10.00
Crude protein, %	23.58	23.58	20.77	22.08	22.08	19.29
Lysine, %	1.41	1.41	1.52	1.13	1.13	1.26
Methionine, %	0.53	0.53	0.55	0.51	0.51	0.51
Crude fat, %	4.16	4.16	5.68	4.05	4.05	5.57
Fiber, %	4.20	4.20	3.81	5.03	5.03	4.64
Starch, %	33.00	33.00	33.97	29.70	29.70	30.67
Calcium	1.06	1.06	1.38	3.31	3.31	3.63
Phosphorus	0.65	0.65	0.66	0.62	0.62	0.63

**Table 2:** Total saturated, monounsaturated, polyunsaturated, and trans fatty acids (%) and fatty acid ratios in feed mixtures for laying quail.

Variables	Feed Mixture for Laying Quail		
	Basal Diet	Resveratrol Suppl.	BSF Suppl.
Saturated fatty acids (SFAs)	17.73	16.36	27.39
Monounsaturated fatty acids (MUFAs)	27.47	26.33	24.19
Polyunsaturated fatty acids (PUFAs)	54.41	57.07	48.41
PUFA/SFA	3.07	3.49	1.77
n-6/n-3 PUFA	19.26	20.93	18.50
*Trans* fatty acids (TFAs)	0.28	0.21	0.12

**Table 3:** Chemical composition (g/kg) and metabolizable energy (MJ/kg) of the defatted black soldier fly larvae meal (BSF).

Items	Amount
Dry matter	884.20
Crude protein	233.20
Crude fat	33.80
Fiber	30.80
Ash	77.80
Calcium	35.80
Phosphorus	8.09
Lysine	38.90
Methionine	12.60
Tryptophan	5.80
Threonine	27.80
Valine	39.60
Isoleucine	30.30
Arginine	33.00
Gross energy	16.44
Metabolizable energy	4.78

**Table 4:** Sociodemographic characteristics of respondents.

Categories	Sociodemographic Factors	*n*	%
Gender	Female	89	67.4
	Male	43	32.6
Generation/Age	Silent (1928–1945)	1	0.8
	Boomers (1946–1964)	32	24.2
	X (1965–1980)	45	34.1
	Millennials Y (1981–1996)	31	23.5
	Z (1997–2012)	23	17.4
Education	Basic	6	4.5
	Secondary	20	15.2
	Special secondary	17	12.9
	Higher	89	67.4

**Table 5:** Effect of dietary treatment on the productive performance of laying quails.

Variables		Dietary Treatment			SED	*p*-Value
		Basal Diet	Resveratrol Suppl.	BSF Suppl.		
Number of quails		50	50	50		
Number replicated cages		9	9	9		
Initial live weight, g		339.7	344.3	346.3	10.880	0.816
Final live weight, g		405.4 ^b^	399.7 ^b^	442.9 ^a^	8.877	0.045
Mortality, %	Month 1	0.00	0.00	0.13	0.072	0.128
	Month 2	0.00 ^b^	0.27 ^a^	0.00 ^b^	0.133	0.065
	Month 3	0.14	0.22	0.07	0.138	0.539
	Months 1–3	0.05	0.16	0.06	0.048	0.188
Egg production, %	Month 1	76.16	73.13	70.63	7.009	0.735
	Month 2	97.58 ^e^	92.04 ^f^	97.19 ^e^	1.240	<0.001
	Month 3	98.68 ^a^	95.68 ^b^	96.13 ^b^	1.177	0.026
	Months 1–3	90.81	86.80	88.08	2.880	0.365
Defective eggs, %	Month 1	5.99	6.01	5.10	2.489	0.920
	Month 2	0.29	0.75	0.10	0.345	0.158
	Month 3	0.00	0.61	0.47	0361	0.219
	Months 1–3	2.09	2.49	1.84	0.919	0.771

Month—period of 30 days past start of laying in which eggs were counted; SED—standard error of difference. Mean values with different superscripts of GLM LSD tests for the treatment groups differ significantly: ^a,b^ *p* < 0.05; ^e,f^ *p* < 0.001.

**Table 6:** Effect of dietary treatment on quail egg morphology.

Variables	Treatment Groups			SED	*p*-Value
	Basal Diet *n* = 102	Resveratrol Suppl. *n* = 102	BSF Suppl. *n* = 102		
Egg weight, g	13.83 ^a^	13.28 ^b^	13.38	0.234	0.048
Egg height, mm	34.64	34.38	34.31	0.359	0.621
Egg diameter, mm	26.72	26.57	26.70	0.179	0.652
Egg shape index	77.21	77.36	77.92	0.829	0.665
Boiled egg weight, g	13.55	13.00	13.10	0.235	0.055
Shell weight, g	1.20	1.17 ^b^	1.26 ^a^	0.036	0.039
Shell percentage %	8.87 ^d^	9.00 ^b^	9.63 ^a,c^	0.252	0.007
Yolk weight, g	3.98	3.81	3.96	0.105	0.207
Yolk percentage %	29.32	29.25	30.24	0.506	0.099
Albumen weight, g	7.84 ^a^	7.60 ^a^	7.41 ^b^	0.155	0.023
Albumen percentage, %	57.91 ^a^	58.43 ^c^	56.55 ^b,d^	0.531	0.002

SED—standard error of difference. Mean values with different superscripts of GLM LSD tests for the treatment groups differ significantly: ^a,b^ *p* < 0.05; ^c,d^ *p* < 0.01.

**Table 7:** Effect of dietary treatment on egg yolk pH and colour parameters.

Variables	Treatment Groups			SED	*p*-Value
	Basal Diet *n* = 17	Resveratrol Suppl. *n* = 17	BSF Suppl. *n* = 17		
pH	6.47 ^e,c^	6.35 ^f^	6.39 ^d^	0.027	0.001
Colour L*	87.55	86.74 ^b^	88.22 ^a^	0.508	0.020
a*	−5.68	−5.71	−5.51	0.205	0.563
b*	39.28	36.81 ^d^	41.47 ^c^	1.413	0.007
C	39.82	37.32 ^d^	41.84 ^c^	1.386	0.008
h	98.35	98.97 ^a^	97.61 ^b^	0.511	0.038

SED—standard error of difference; L*—lightness; a*—redness/greenishness; b*—yellowness; C—chroma; h—hue angle.Meanvalues with different superscripts of GLM LSD tests for the treatment groups differ significantly: ^a,b^ *p* < 0.05; ^c,d^ *p* < 0.01; ^e,f^ *p* < 0.001.

**Table 8:** Effect of dietary treatment on proximate composition of boiled quail eggs.

Variables	Treatment Groups			SED	*p*-Value
	Basal Diet *n* = 15	Resveratrol Suppl. *n* = 15	BSF Suppl. *n* = 15		
Yolk					
Dry matter,%	51.03	51.42	51.35	0.176	0.073
Protein,%	15.64 ^f^	16.03 ^e^	15.54 ^f^	0.100	<0.001
Lipids,%	28.73 ^c^	26.85	24.66 ^d^	1.232	0.008
Ash,%	1.73 ^d^	1.84	1.94 ^c^	0.057	0.003
Cholesterol, mg/g	11.28	11.20	10.69	0.298	0.109
Albumen					
Dry matter,%	14.46 ^d,f^	14.72 ^c^	14.80 ^e^	0.076	<0.001
Protein,%	12.53 ^f^	12.69 ^f^	13.35 ^e^	0.087	<0.001
Lipids,%	0.39 ^e^	0.30 ^f^	0.28 ^f^	0.020	<0.001
Ash,%	0.66 ^d,f^	0.73 ^c^	0.80 ^e,d^	0.020	<0.001
Edible portion					
Dry matter,%	25.18 ^b^	25.58	26.17 ^a^	0.325	0.014
Protein,%	13.21	13.32	13.41	0.277	0.786
Lipids,%	9.75	9.82	9.84	0.382	0.972
Ash,%	0.96 ^a^	0.80 ^b,f^	1.04 ^e^	0.060	0.001

SED—standard error of difference. Mean values with different superscripts of GLM LSD tests for the treatment groups differ significantly: ^a,b^ *p* < 0.05; ^c,d^ *p* < 0.01; ^e,f^ *p* < 0.001.

**Table 9:** Effect of dietary treatment on fatty acid profile in yolk lipids of quail.

Fatty Acids	Treatment Groups			SED	*p*-Value
	Basal Diet *n* = 15	Resveratrol Suppl. *n* = 15	BSF Suppl. *n* = 15		
C12:0	0.06	0.05 ^d^	0.08 ^c^	0.009	0.009
C14:0	0.37 ^f^	0.33 ^f^	0.68 ^e^	0.025	<0.001
C15:0	0.03	0.03	0.03	0.002	0.151
C16:0	26.27	25.95	26.82	0.377	0.078
C17:0	0.10 ^b,f^	0.12 ^a^	0.13 ^e^	0.006	<0.001
C18:0	7.84	8.20	8.22	0.173	0.056
C20:0	0.02	0.02	0.02	0.003	0.141
C21:0	0.03	0.03	0.03	0.003	0.912
C22:0	0.14 ^c,a^	0.11 ^d^	0.12 ^b^	0.009	0.003
SFA	34.87 ^b^	34.83 ^d^	36.13 ^a,c^	0.407	0.003
C14:1n-7	0.08 ^f^	0.06 ^f^	0.18 ^e^	0.010	<0.001
C16:1n-7trans	0.02	0.02	0.02	0.002	0.358
C16:1n-9	0.50 ^a^	0.57 ^e^	0.42 ^b,f^	0.030	0.001
C16:1n-7	4.66 ^c^	3.94 ^d,b^	4.53 ^a^	0.212	0.004
C17:1n-9	0.04 ^b,d^	0.06 ^a^	0.06 ^c^	0.005	0.004
C18:1n-9*trans*	0.13	0.12	0.12	0.005	0.159
C18:1n-9	42.53	43.58	42.63	0.762	0.321
C18:1n-7	2.27 ^e^	2.10	1.95 ^f^	0.076	0.001
C20:1n-9	0.45	0.43	0.40	0.005	0.600
MUFA	49.76	49.93	49.53	0.745	0.862
C18:2n-6*trans*	0.03	0.03	0.04	0.006	0.680
C18:2 n-6c,*t*	0.06 ^e^	0.05^c^	0.04 ^f,d^	0.004	0.001
C18:2 n-6*t*,c	0.04 ^a^	0.03	0.03 ^b^	0.005	0.028
C18:2 n-6	10.77	10.64	10.24	0.534	0.598
C18:3 n-6	0.14 ^a^	0.13	0.11 ^b^	0.010	0.046
C18:3 n-3	0.36 ^a^	0.34	0.30 ^b^	0.021	0.019
C20:2 n-6	0.03 ^a^	0.03 ^a^	0.02 ^b^	0.002	0.004
C20:3 n-6	0.15 ^c,a^	0.12 ^d^	0.13 ^b^	0.009	0.004
C20:4 n-6	1.20	2.10	1.92	0.082	0.078
C20:5 n-3	0.04 ^d^	0.05	0.06 ^c^	0.007	0.008
C22:4 n-6	0.11 ^c^	0.11^c^	0.09 ^d^	0.007	0.002
C22:5 n-3	0.09 ^c^	0.08	0.07 ^d^	0.007	0.006
C22:6 n-3	0.52 ^e^	0.48 ^a^	0.40 ^f,b^	0.030	0.001
PUFA	14.33	14.20	13.44	0.579	0.263
TFA	0.28 ^c^	0.25	0.24 ^d^	0.011	0.004
UFA	0.53	0.47	0.49	0.036	0.201

SED—standard error of difference; SFA = sum of all identified saturated fatty acids; MUFA = sum of all identified monounsaturated fatty acids; PUFA = sum of all identified polyunsaturated fatty acids; TFA = sum of all identified *trans* fatty acids; UFA—sum of all unidentified fatty acids.Mean values with different superscripts of GLM LSD tests for the treatment groups differ significantly: ^a,b^ *p* < 0.05; ^c,d^ *p* < 0.01; ^e,f^ *p*< 0.001.

**Table 10:** Effect of dietary treatment on fatty acid ratios and lipid quality indexes in yolk lipids of quail eggs.

Variables	Treatment Groups			SED	*p*-Value
	Basal Diet	Resveratrol Suppl.	BSF Suppl.		
PUFA/SFA	0.41 ^a^	0.41 ^a^	0.37 ^b^	0.016	0.037
n-6/n-3	13.28 ^d^	13.91	15.30 ^c^	0.642	0.010
AI	0.44 ^f^	0.43 ^f^	0.47 ^e^	0.007	<0.001
TI	1.00 ^f^	1.00 ^f^	1.06 ^e^	0.017	<0.001
h/H	2.22 ^a^	2.28 ^e^	2.11 ^b,f^	0.035	0.004
HPI	2.31 ^e^	2.35 ^e^	2.13 ^f^	0.048	<0.001
FLQ	1.60 ^e^	1.54 ^e^	1.28 ^f^	0.092	0.003
PI	26.84 ^a^	26.74 ^a^	24.70 ^b^	0.822	0.020

PUFA/SFA—ratio of PUFA to SFA; n-6/n-3—ratio of n-6 PUFA to n-3 PUFA; AI—atherogenic index; TI—thrombogenic index; h/H—hypocholesterolemic/hypercholesterolemic ratio; HPI—health-promoting index; FLQ—flesh (yolk) lipid quality index; PI—peroxidisability index. Mean values with different superscripts of GLM LSD tests for the treatment groups differ significantly: ^a,b^ *p* < 0.05; ^c,d^ *p* < 0.01; ^e,f^ *p* < 0.001.

**Table 11:** Effect of dietary treatment on yolk oxidative status in relation to MDA (μmol MDA/1 kg of yolk) content.

Variables	Treatment Groups			SED	*p*-Value
	Basal Diet *n* = 10	Resveratrol Suppl. *n* = 10	BSF Suppl. *n* = 10		
Day 0	0.192 ^F^	0.168 ^F^	0.146 ^F^	0.019	0.065
Day 30	0.422 ^a,E^	0.353 ^E^	0.317 ^b,E^	0.041	0.040
Day 60	0.421	0.361	0.401	0.030	0.176

SED—standard error of difference; MDA—malondialdehyde. Mean values with different superscripts of GLM LSD tests for the treatment groups in the rows differ significantly: ^a,b^ *p* < 0.05; and in the columns ^E,F^ *p* < 0.001.

**Table 12:** Effect of dietary treatment and consumer gender and generation on sensory acceptance of quail eggs.

Variables	Treatment Groups			SED	Consumer Gender		SED	*p*-Value		
	Basal Diet	Resveratrol Suppl.	BSF Suppl.		F	M		Treatment Group	Gender	Generation
Odour	6.96	7.14	7.21	0.254	6.99	7.21	0.208	0.583	0.282	0.178
Colour intensity of yolk	6.96 ^d^	7.04 ^d^	7.62 ^c^	0.219	7.18	7.23	0.178	0.005	0.777	0.135
Flavour	7.18 ^d^	7.61	7.78 ^c^	0.184	7.52	7.54	0.150	0.004	0.875	0.592
Overall acceptability of egg	7.25 ^b^	7.56	7.67 ^a^	0.175	7.50	7.49	0.143	0.043	0.956	0.444

F—female; M—male; SED—standard error of difference. Mean values with different superscripts of GLM LSD tests for the treatment groups differ significantly: ^a,b^ *p* < 0.05; ^c,d^ *p* < 0.01.

**Table 13:** Emotional response to eggs from quail fed with different diets.

	Emotions	Treatment Groups			SED	*p*-Value
		Basal Diet	Resveratrol Suppl.	BSF Suppl.		
View	Neutral	0.597	0.619	0.616	0.065	0.935
	Happy	0.004	0.010	0.009	0.009	0.758
	Sad	0.080	0.068	0.138	0.059	0.464
	Angry	0.022	0.014	0.029	0.021	0.776
	Surprised	0.003	0.009	0.001	0.005	0.299
	Scared	0.001	0.004	0.003	0.004	0.755
	Disgusted	0.064	0.071	0.057	0.054	0.966
	Contempt	0.006	0.001	0.005	0.003	0.323
	Valence	−0.146	−0.129	−0.198	0.042	0.258
Odour	Neutral	0.638	0.632	0.621	0.066	0.968
	Happy	0.003	0.025	0.000	0.013	0.158
	Sad	0.061	0.046	0.045	0.024	0.765
	Angry	0.043	0.022	0.048	0.023	0.473
	Surprised	0.009	0.017	0.001	0.014	0.560
	Scared	0.002	0.001	0.002	0.002	0.971
	Disgusted	0.035	0.026	0.041	0.020	0.777
	Contempt	0.010	0.004	0.002	0.005	0.293
	Valence	−0.110	−0.053	−0.098	0.025	0.096
Taste	Neutral	0.649	0.610	0.652	0.067	0.783
	Happy	0.018	0.063	0.005	0.044	0.412
	Sad	0.114	0.088	0.069	0.048	0.653
	Angry	0.044	0.037	0.093	0.027	0.103
	Surprised	0.004	0.009	0.009	0.007	0.726
	Scared	0.001	0.006	0.009	0.008	0.645
	Disgusted	0.031	0.028	0.026	0.012	0.935
	Contempt	0.012	0.009	0.008	0.005	0.700
	Valence	−0.137	−0.068	−0.164	0.070	0.395

## Data Availability

The original contributions presented in the study are included in the article; further inquiries can be directed to the corresponding author.

## Supplementary Materials

The following supporting information can be downloaded at: https://www.mdpi.com/article/10.3390/ani15010042/s1, Table S1: Egg numbers used for quality evaluation.
