# Supplementary material for: Effects of Dietary Resveratrol and Black Soldier Fly (Hermetia illucens) Larvae Meal Supplements on Quail Egg Production, Quality, and Consumer Acceptance
Source: Animals (Basel). 2024 Dec 27;15(1):42. doi: 10.3390/ani15010042 (PMC11718990; doi:10.3390/ani15010042)
Supplement: Supplementary file 1 [file animals-15-00042-s001.zip › animals-3330769-supplementary.pdf]

Table S1. Egg numbers used for quality evaluation.

	Eggs per sample	No of samples	No of eggs/treatment	Total number of eggs	Week post the start of laying
External and internal characteristics	1	102	102	306	6
pH and colour of yolk (homogenized)	6	17			
Chemical composition and yolk lipid profile analyses (homogenized)	8	15	120	360	7
Cholesterol content	1	15	15	45	7
Storage stability of egg (MDA)	1	10	30	90	8
Consumer sensory evaluation	2	132	264	792	9-10
Evaluation of emotional response to egg			30	90	9
Total number of eggs used			561	1683	6-10
